# A comparison of tiotropium/olodaterol vs tiotropium alone in terms of treatment effect for chronic obstructive pulmonary disease: A meta-analysis: Retraction

**DOI:** 10.1097/MD.0000000000022420

**Published:** 2020-09-18

The article “A comparison of tiotropium/olodaterol vs tiotropium alone in terms of treatment effect for chronic obstructive pulmonary disease: A meta-analysis”,^[1]^ which appeared in Volume 99, Issue 16 of *Medicine* is being retracted after an investigation of reader concerns. The concerns, published on the *Medicine Correspondence Blog*,^[2]^ detail a misrepresentation of data as post-treatment efficacy data and the inappropriate use of selected studies in the meta-analysis. Authors were unresponsive in providing corrections to address the raised concerns.
